# Dual Role of CD4 in Peripheral T Lymphocytes

**DOI:** 10.3389/fimmu.2019.00618

**Published:** 2019-04-02

**Authors:** Daniela Glatzová, Marek Cebecauer

**Affiliations:** ^1^Department of Biophysical Chemistry, J. Heyrovsky Institute of Physical Chemistry of the Czech Academy of Sciences, Prague, Czechia; ^2^Laboratory of Leukocyte Signaling, Institute of Molecular Genetics of the Czech Academy of Sciences, Prague, Czechia

**Keywords:** T lymphocytes, CD4, TCR coreceptor, Lck, cell-cell adhesion, microvilli

## Abstract

The interaction of T-cell receptors (TCRs) with self- and non-self-peptides in the major histocompatibility complex (MHC) stimulates crucial signaling events, which in turn can activate T lymphocytes. A variety of accessory molecules further modulate T-cell signaling. Of these, the CD4 and CD8 coreceptors make the most critical contributions to T cell sensitivity *in vivo*. Whereas, CD4 function in T cell development is well-characterized, its role in peripheral T cells remains incompletely understood. It was originally suggested that CD4 stabilizes weak interactions between TCRs and peptides in the MHC and delivers Lck kinases to that complex. The results of numerous experiments support the latter role, indicating that the CD4-Lck complex accelerates TCR-triggered signaling and controls the availability of the kinase for TCR in the absence of the ligand. On the other hand, extremely low affinity of CD4 for MHC rules out its ability to stabilize the receptor-ligand complex. In this review, we summarize the current knowledge on CD4 in T cells, with a special emphasis on the spatio-temporal organization of early signaling events and the relevance for CD4 function. We further highlight the capacity of CD4 to interact with the MHC in the absence of TCR. It drives the adhesion of T cells to the cells that express the MHC. This process is facilitated by the CD4 accumulation in the tips of microvilli on the surface of unstimulated T cells. Based on these observations, we suggest an alternative model of CD4 role in T-cell activation.

## Introduction

**Figure 1:**
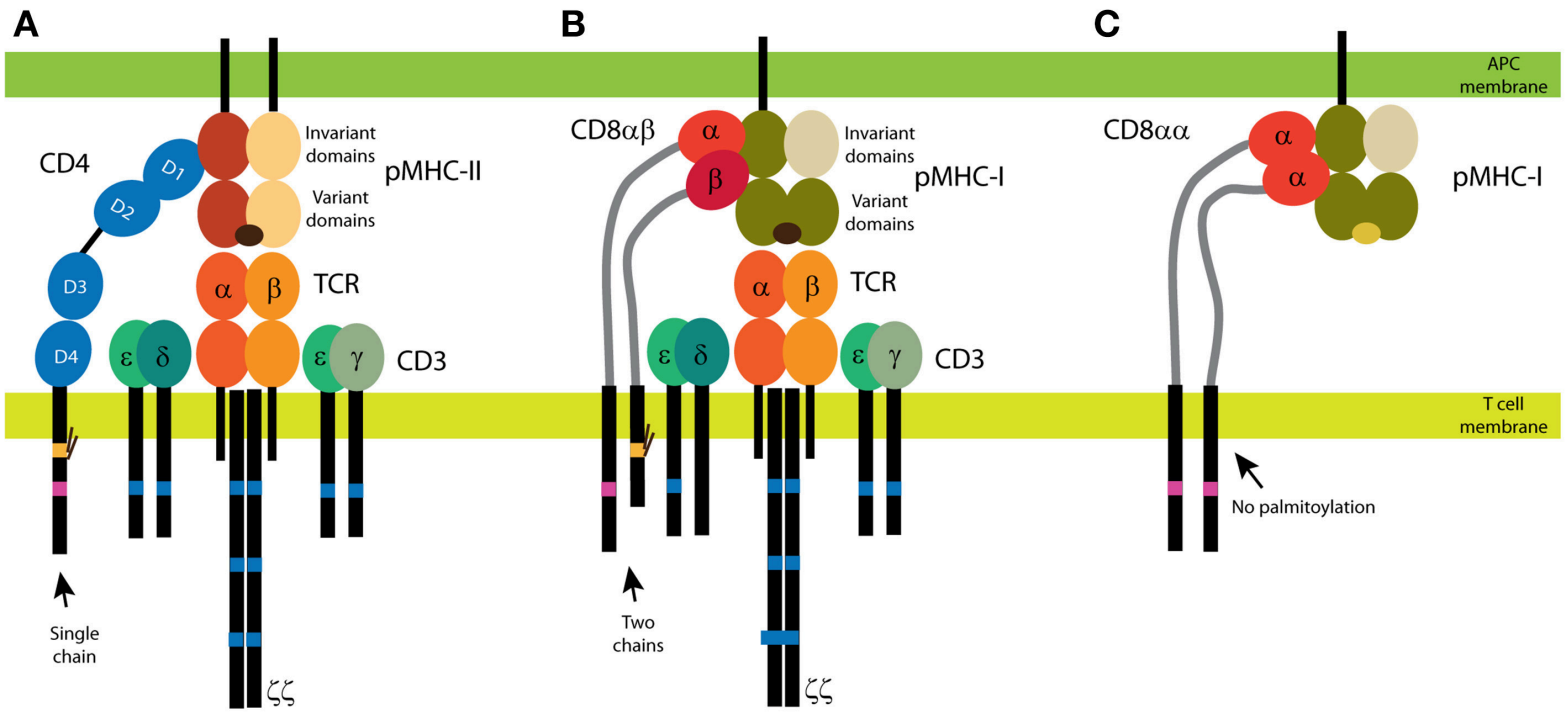
CD4 and CD8 coreceptors. **(A)** The CD4 glycoprotein is composed of a single chain. Its functional motifs, such as the Lck-binding site (in magenta) and the palmitoylation site (in yellow), are in the sole intracellular domain. The extracellular part of CD4 is composed of four Ig-like domains, and the MHC binding site is in the N-terminal D1 domain. Short linker connects CD4 extracellular domains with the transmembrane domain. **(B,C)** Two forms of CD8 exist: the αβ heterodimer **(B)** and the αα homodimer **(C)**. The α subunit of CD8 contains the Lck-binding site, and the β subunit contains the palmitoylation site. A single Ig-like domain and a long stalk region (in light gray) form the extracellular parts of the CD8 subunits. Binding of CD4 **(A)** and CD8αβ **(B)** to MHC is illustrated with the antigenic receptor because these coreceptors support receptor function in T cells. The TCR/CD3 complex is composed of at least eight subunits. CD3 subunits γ, δ, and ε contain one immunoreceptor tyrosine-based activation motif (ITAM; in dark blue) and three ITAMs are in each ζ subunit. Cognate peptides are depicted in dark brown, self-antigens in light brown.

In vertebrates, T lymphocytes (also called T cells) continuously scan tissues for foreign antigens. On the surface of these cells, T-cell receptors (TCRs) recognize the antigens as short peptides bound to the major histocompatibility complex (MHC) on antigen-presenting cells (APCs; Figure 1). Thus, TCR-peptide-MHC (pMHC) pairs determine the specificity of the T cell-dependent immune response. However, several other surface receptors of T cells (e.g., CD2, CD4, CD5, CD8, CD28, and LFA-1) and of APCs (e.g., CD58, CTLA-4, and ICAM-1) can regulate the sensitivity and output of T cell responses. Of these receptors, CD4 and CD8 most critically contribute to the T cell function *in vivo* and thus are known as coreceptors of TCR. CD4 and CD8 share ligands with TCRs by binding to invariant segments of the MHC (Figure 1). As discussed below, CD4 and CD8 also contribute to T-cell development, homeostasis and antigenic response. However, the mechanisms behind these activities are not yet fully understood. This is especially true for CD4, which has extremely low affinity for its ligand but which is also essential in T-cell development and in the removal of pathogens during T cell-dependent immune responses.

In this work, we focus on dual role of CD4 in peripheral T cells. Contributions of CD4 to antigen-dependent TCR signaling are well-established. However, its antigen-independent function has not been studied in detail. After a brief introduction to the biochemistry of initial events, we focus on providing more in-depth insight into the spatio-temporal organization of signaling events in T cells so as to highlight the importance of nanoscopic localization of molecules. In later sections, we present and discuss the accumulated knowledge on function of CD4 in TCR signaling, with an emphasis on spatial organization of CD4 in T cells. Finally, we describe antigen-independent role of CD4 and speculate on its role in T-cell activation.

## T Cells and Antigen-induced Signaling

T cells originate in bone-marrow haematopoietic stem cells. The progenitors of these cells migrate to the thymus, where thymocytes undergo a series of maturation and selection processes to complete the TCR expression and to avoid stimulation by self-antigens. This process, called thymic T cell development, gives rise to the peripheral pool of T cells, which mainly express αβTCR. Although 1–10% of T cells express γδTCR on their surface, these cells recognize non-peptidic antigens (1). This review focuses on peripheral αβ T cells.

TCRs are heterodimers formed by the subunits α and β, each of which contains two extracellular immunoglobulin (Ig)-like domains, a single transmembrane domain and a short intracellular tail that lacks any known structural or functional motif (Figure 1). The αβ heterodimer forms a complex with the CD3 subunits (γ, δ, ε, ζ) for surface expression and full function (Figure 1). The intracellular tails of CD3 subunits contain immunoreceptor tyrosine-based activation motifs (ITAMs), which are involved in TCR-induced signaling. The TCR/CD3 complex lacks enzymatic activity. This distinguishes TCRs (and other immunoreceptors) from the receptors that directly stimulate downstream events upon binding to a ligand (e.g., receptor kinases).

Based on the current understanding of these processes, it is predicted that the interaction between TCRs and the pMHC is the first step toward antigen-induced T-cell activation. Consequently, early signaling events can be detected when Lck kinase phosphorylates ITAMs in the cytosolic tails of the CD3 subunits that are associated with TCR. Each ITAM contains two phosphorylated tyrosines, which serve as high-affinity docking sites for the tandem SH2 domains of ZAP-70 kinase. Lck also phosphorylates and binds ZAP-70 to induce its full activation (2). As Lck is bound to ZAP-70 via its SH2 domain, its open form provides a docking site (the SH3 domain) for the LAT adaptor protein. This leads to bridging between ZAP-70 and its substrates, LAT and SLP-76 (3). The ZAP-70 phosphorylation of the activating tyrosines on LAT forms a platform for the interactions of LAT with signaling molecules such as SLP-76, Grb2/Sos, PLCγ1, and Vav1, and for the formation of a signalosome that regulates the downstream effector events associated with T-cell activation (4). Although the signaling pathways that are downstream of LAT have been thoroughly described, the initial events of the T-cell activation are still incompletely understood (5). Importantly, there is a lack of clarity regarding how and when Lck associates with the TCR-signaling complex. CD4 and CD8 potentially play important roles in this process because, in resting T cells, a large fraction of Lck is associated with these molecules (6, 7).

## T-cell Activation Events: Spatiotemporal Organization

**Figure 2:**
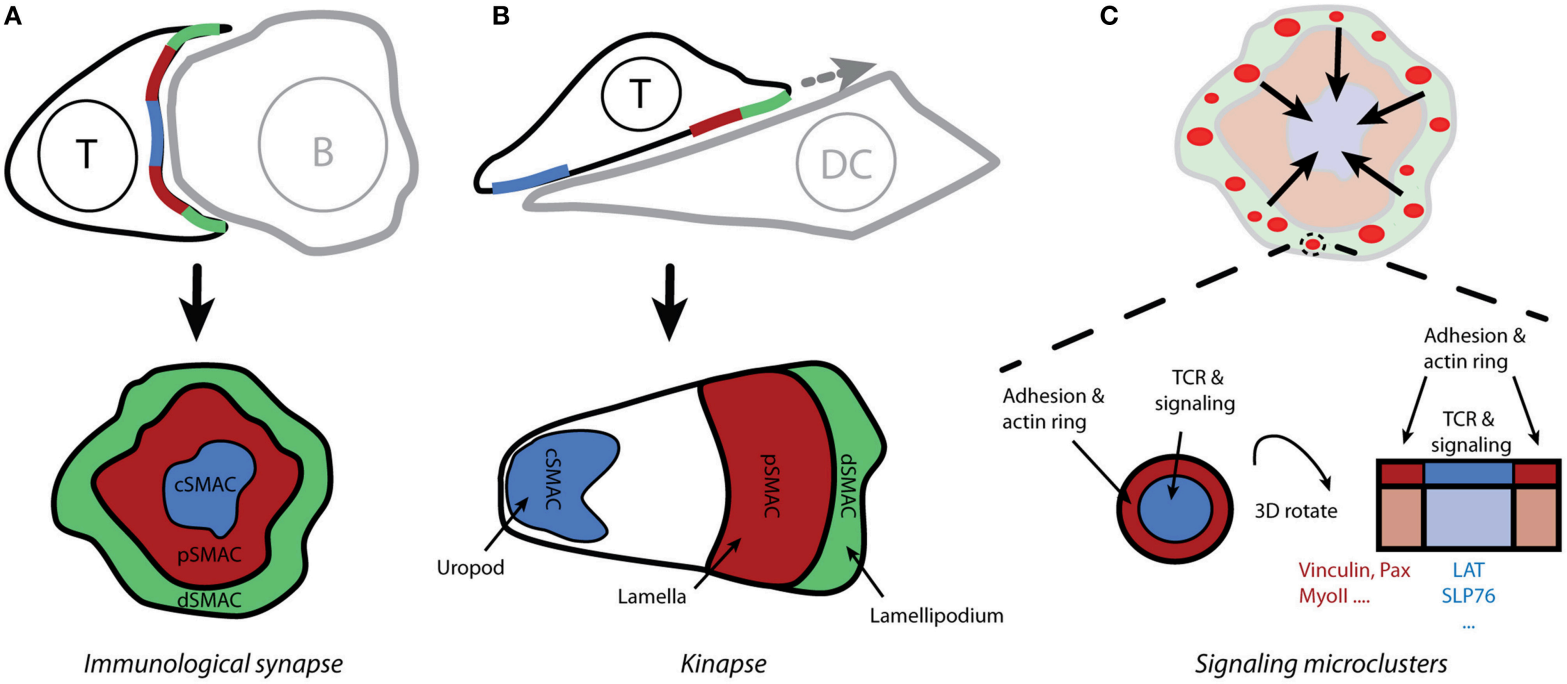
Spatio-temporal organization of T-cell activation. **(A)** The contact site between T cells and antigen-presenting cells (which form conjugates) is called the immunological synapse due to its similarity to neuronal synapses. Live-cell confocal microscopy reveals the supramolecular organization of molecules within the immunological synapse, with the receptors and effector molecules accumulating in the center of the mature synapse (cSMAC) and adhesion molecules forming a peripheral ring (pSMAC; i.e., the bull's eye model). **(B)** Motile T cells form asymmetric kinapses instead of stable and symmetric synapses. Kinapses are similar to motile fibroblast cells with lamellipodium (dSMAC), lamella (pSMAC), and uropod (cSMAC). **(C)** Total internal-reflection fluorescence microscopy reveals that T-cell signaling is initiated in small microclusters that are assembled upon antigenic stimulation in actin-rich distal regions of the immunological synapse (**A**; dSMAC). Small rings of adhesion molecules and actin surround TCR microclusters and could serve to stabilize those microclusters.

After recognizing an antigen, T cells form tight contact with target cells. The instruction to stop the crawling of T cells comes from the interaction of TCRs with a cognate pMHC. This process, which is called inside-out signaling, enhances integrin affinity for its ligands [e.g., ICAM-1; (8, 9)]. Consequently, a site of extensive contact between T cells and APC forms. The contact site remains dynamic but is also highly organized in time and space. As such sites are reminiscent of the synapses formed between neurons they are named *immunological synapses* [IS; Figure 2; (10, 11)]. In addition to TCRs and pMHC, diverse stimulatory and inhibitory receptor-ligand pairs, as well as intracellular signaling molecules are localized in the IS during T-cell activation (4).

In the classical model of IS, the TCR receptor and signaling molecules accumulate in the center of a structure that is reminiscent of a bull's eye (Figure 2A). This area is called the central supramolecular activating cluster (cSMAC) and it is surrounded by an adhesive ring (LFA-1/ICAM-1) called a peripheral SMAC (pSMAC). The initial theory was that an IS functions as a stabilizing element, supporting sustained signaling via TCRs (12). Researchers challenged this concept after finding that T cells often lack a classical IS when conjugated with dendritic cells that are loaded with physiological levels of antigen (13). Several observations indicate that IS result from, rather than being a prerequisite for, T-cell signaling (14–17). It is thus evident that, at least in its early phase, the IS structure must be more dynamic than originally thought; this resulted in the concept of *kinapses* (18). When forming kinapses (Figure 2B), migrating T cells decelerate upon stimulation but do not stop to form stable, symmetric synapses. The T-cell leading edge, which is reminiscent of the lamellipodium in motile fibroblasts, forms a structure for TCR signaling initiation, whereas adhesive molecules and a densely branched actin cytoskeleton accumulate in the lamella. Importantly, the signaling and the adhesive molecules are spatially segregated in both modes of the T cell-APC contact site: synapses and kinapses. Primary T cells mainly form kinapses when interacting with stimulatory cells or surfaces, both *in vivo* and *in vitro* (17, 19).

Whereas, intravital microscopy confirms the formation of organized contact sites between T cells and APCs *in vivo*, a better understanding of spatio-temporal organization of signaling events required new microscopy techniques (e.g., total internal-reflection fluorescence and super-resolution microscopy) and supported planar bilayers functionalized with activating (pMHC) and adhesive (ICAM-1) molecules (20, 21). Improved imaging conditions enabled the discovery of *signaling microclusters*. For instance, TCR microclusters are formed in the distal regions of the IS (Figures 2A,C) and represent the sites of signal initiation (22, 23). These microclusters are associated with essential signaling components such as Lck, ZAP-70 and LAT, but they exclude CD45 phosphatase, which can dephosphorylate ITAMs (2, 15, 23). Interestingly, TCR microclusters are mobile assemblies. In response to strong antigenic stimulation, the microclusters move centripetally from the periphery toward the center of the IS [Figure 2C; (11, 15, 22)]. It is unclear how these structures stabilize during movement over several microns. The LFA-1/ICAM-1 micro-adhesive rings that surround microclusters, thus forming micro-synapses, may be a stabilizing factor [Figure 2C; (24)]. Of note, signaling microclusters gave way to *nanoclusters* due to improvements in microscopes, which provide more appropriate information about the size of these clusters: 100-500 nm (25–27).

The Varma group reported the existence of TCR microclusters in unstimulated T cells (28) and found that the number of TCR in microclusters remains constant upon stimulation. The LAT and Grb2 signaling molecules are associated with the pre-existing clusters, and CD45 is excluded from these structures even before the antigen stimulation (28). These results indicate that TCRs and effector molecules are pre-assembled in structures that are unresolvable using standard light microscopy [<300 nm; (29)]. Moreover, the data on the molecular organization of TCR microclusters are limited. Some researchers have reported the existence of TCR oligomers on the surface of T cells before antigen stimulation (30, 31), but others demonstrated that TCRs have random distribution and a monomeric character during ligand recognition (32–34). TCR assembly in higher-order structures then occurs upon stimulation (34). Importantly, pre-assembling of the receptor and the effector signaling molecules in higher-order structures could explain the rapid responsiveness of T cells (35). However, it is not clear whether these models authentically represent the T cells in the tissues of higher vertebrates. Current imaging technologies do not allow for high-resolution imaging of cellular structures in living animals.

## T Cell Coreceptors

The previous sections focus on the essential molecules involved in T-cell activation and on descriptions of the morphological and molecular structures which were previously found to contribute to this process. These studies have usually investigated TCRs and downstream signaling molecules; they thus have provided little information about the involvement of coreceptors. The experiments have been often performed using stimulation with anti-CD3 or TCR antibodies, which overpass CD4 and CD8 coreceptors in the initial phase of signaling. Such simulation activates T cells, as determined by the IL-2 production and increased expression of the CD25 and CD69 activation markers (36).

*In vivo*, CD4 and CD8 are essential for proper T cell development and thymic selection. These two coreceptors control the MHC specificity of selected thymocytes by limiting availability of Lck for TCR signaling in the absence of the ligand binding (6, 37). In peripheral T cells, the expression of coreceptors is mutually exclusive. CD4^+^ T cells primarily provide help for B lymphocytes and innate immune cells during infections, whereas most CD8^+^ T cells exhibit cytotoxicity toward virally infected or tumor cells. However, this definition is insufficient because the periphery contains many subsets of T cells with highly specific functions (38). In this review, we focus on general role of CD4 in T-cell activation, irrespective of cell type. We are aware that the coreceptor levels vary in T-cell subsets and that this can affect CD4 function (39). However, there is insufficient data to elaborate on specific function of CD4 in all CD4^+^ T-cell subsets. Sewell and colleagues reviewed CD8 and its function in T cells (40); please also see the direct comparison of CD4 and CD8 coreceptors in Box 1.

Box 1 **The CD4 and CD8 Coreceptors Are Structurally Diverse.**

Even though both are called coreceptors, CD4 and CD8 have significantly different expression profiles and structures. CD8 is a dimer that occurs in two forms: the CD8αα homodimer and the CD8αβ heterodimer (Figure 1). It is predominantly expressed in a subset of T cells, but it can be found in some natural killer and dendritic cells (41–43). Little is known about the function of the CD8αα homodimer (44). The CD8αβ heterodimer supports TCR signaling when stimulated by antigens on MHC class I (45). In the heterodimer, two subunits with two intracellular tails can modulate TCR activity. Each subunit of CD8 also contains a single globular, Ig-like domain in the N-terminus; this domain is linked to the transmembrane domain via a long, flexible stalk. By contrast, CD4 comprises a single chain [Figure 1; (46)]. Its single intracellular part defines all functions in the downstream signaling. Moreover, CD4 extracellular part is composed of four globular, Ig-like domains that are linked to the transmembrane domain only via a few amino-acid residues. Thus, CD4 extracellular part is extended further from the T-cell membrane and exhibits less flexibility, as compared to the extracellular part of CD8. Both CD4 and CD8 coreceptors can be palmitoylated and can bind Lck. In CD8, the palmitoylation site is in the β subunit (45), and the Lck-binding site is in the α subunit (47, 48). Both motifs are in single cytoplasmic tail of CD4 [Figure 1; (47–49)]. These structural differences indicate that T cells use the specific properties of CD4 and CD8 to fine-tune their physiological roles.

## Structure and Function of CD4

Extracellular domain of CD4, which is responsible for the recognition of its ligands, is composed of four globular Ig-like domains (D1-D4; Figure 1). Whereas, the binding site for IL-16 is in the membrane-proximal D4 domain, the N-terminal D1 domain binds to a segment of the non-polymorphic β2 domain of MHC class II (50, 51). Similarly, HIV (gp120) binds to D1 domain of CD4 (52). Important roles of CD4 in the life cycle of the HIV virus and in the activity of IL-16 in immune responses were reviewed recently (53, 54). To avoid the complexity of herein discussed processes, we focus on CD4 interaction with MHCII in the absence of other ligands.

The intracellular part is responsible for CD4 palmitoylation [residues 419 and 422 in human CD4 according to the UNIPROT numbering; (49)]. This reversible posttranslational modification is supposed to target proteins in lipid microdomains (55). It also contains a basic-rich motif (residues 423-427: sequence RHRRR) and a Lck-binding site (residues 445 and 447). The transmembrane domain of CD4 contains a conserved GGxxG motif, which was reported to mediate the dimerization of membrane proteins (56). However, such effect has not been confirmed for CD4. Rather, the mutation of this motif to GVxxL reduces the capacity of CD4 to enhance T cell sensitivity to weak antigens (57). This indicates that the importance of the CD4 transmembrane domain in T-cell activation but the molecular mechanism remains unknown.

**Figure 3:**
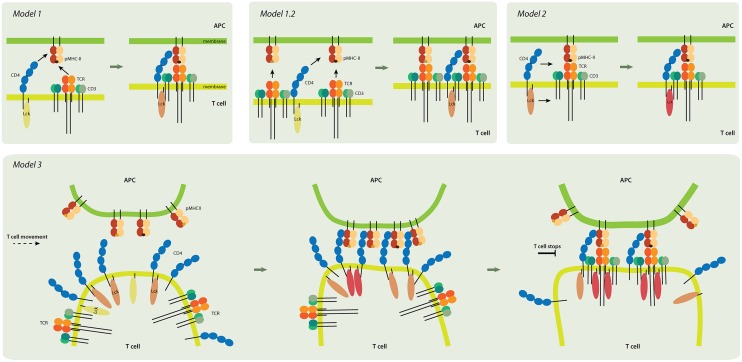
Models of CD4 role in T-cell activation. *Model 1 (upper left panel)*. TCR and CD4 independently interact with pMHC, and the coreceptor stabilizes the receptor-ligand complex. Inactive Lck (yellow) becomes partially activated (light pink). *Model 1.2 (upper middle)*. In this pseudo-dimer model, two TCRs interact with two pMHCs. One MHC always contains an agonist peptide (dark brown), but the other can have a non-stimulating (self) peptide (light brown) instead. The role of CD4 is to crosslink the two receptor-ligand pairs and to thus accelerate signaling. *Model 2 (upper right)*. CD4 delivers the Lck kinase to the formed TCR-pMHC super-complex that has the antigenic peptide. Lck is fully activated (dark pink) by binding to ZAP-70 (not shown) and accommodating open conformation. *Model 3 (lower panel)*. CD4 accumulates at the tips of T-cell microvilli. Independent of TCR, the CD4 that is on microvilli interacts with the pMHC that is assembled on similar membranous protrusions of antigen-presenting cells (APC). This multivalent interaction drives putative changes in the tips of the microvilli and activates Lck; it thus provides T cells and their TCRs with high sensitivity to antigenic peptides. The presented models are not mutually exclusive. Color-coding of CD4, TCR/CD3 complex, and MHCII as in Figure 1.

The coreceptor CD4 is expressed in a subset of T cells, natural-killer (NK) cells, monocytes and macrophages. In macrophages and NK cells, CD4 plays a role in differentiation, migration and cytokine expression (58, 59). In T cells, it is involved in thymic development and antigen recognition in the periphery (46). Although function of CD4 in the thymus is well-known, its role in the activation of peripheral T cells remains enigmatic. Originally, two models of CD4 function in peripheral T cells were suggested: *1)* CD4 stabilizes the ternary complex of pMHC-TCR [***Model 1***; Figure 3 (46)], and *2)* CD4 recruits Lck kinase to the proximity of the TCR/CD3 complex in order to phosphorylate the ITAMs of CD3 molecules and initiate intracellular signaling during antigen-induced T cell activation [***Model 2***; Figure 3; (46, 60–62)].

The interaction of TCRs with pMHCII is CD4-independent. In some cases, as in the presence of a very strong agonist, this interaction can activate T cells (63). However, CD4 is required for the recognition of most antigens *in vivo*. The presence of the CD4 coreceptor enhances T cell sensitivity to antigens by 30- to 100-fold (64–67) and reduces by approximately tenfold the number of antigenic peptides on APCs that are required for sustained TCR signaling (68). Therefore, CD4 is often depicted as a part of the tightly assembled TCR receptor complex, along with agonist pMHCII (Figure 3, Model 1). However, the plasma membrane organization of the CD4-TCR-pMHC assembly remains unknown. In crystallographic studies of the quaternary complex (which comprises the extracellular domains of TCR, pMHCII and CD4), researchers have revealed a V-shaped arch that is created when TCR and CD4 bind simultaneously to the same pMHCII (51). This structure suggests that TCR bound to pMHCII forms one arm of the arch and that CD4 forms the other arm. The CD4-pMHCII contact site appears as the apex of this structure. The geometry of the interacting extracellular domains of pMHCII, TCR and CD4 supports the formation of the quaternary complex (69). However, the lack of the extracellular CD3 domains and other membrane components in the studied complex has led to speculations regarding the CD4-TCR-pMHC assembly under the physiological conditions of two interacting cells.

The Davis group suggested an alternative structure known as the “pseudo-dimer” model [***Model 1.2*** in Figure 3; (70, 71)]. In this model, two TCR-pMHC pairs form a minimal signaling unit and CD4 bridges the two pairs by binding to MHCII, which contains an agonist (antigenic) peptide, as well as by associating with the TCR of the other TCR/MHCII pair, which contains endogenous (self) peptide (71). Importantly, in this model, they attempt to explain extreme sensitivity of CD4^+^ T cells by suggesting that endogenous peptides play a positive role in T-cell activation (70, 71). Most pMHCII on the surface of APCs contain peptides that are derived from endogenous proteins. Only very few antigenic peptides can be found on the MHCII of mature APCs (70, 72). Therefore, T cells must detect rare antigens in a sea of endogenous peptides by adjusting the TCR activation unit toward high sensitivity. However, T cells simultaneously have to distinguish small differences in affinities and/or the kinetics of TCR binding to agonist or self pMHCII (73). Recently, it was found that stimulatory TCR-pMHCII interaction involves numerous catch bonds; no such bonds exist for interactions that do not involve stimulation (74). This observation provides a new explanation for the numerous exceptions to the rule that 3D affinity of TCR for antigenic pMHCII is five- to seven-fold stronger than its affinity for ligands with endogenous peptides. On the other hand, this finding does not explain how such minor differences in TCR-pMHC binding lead to opposite outputs in T cells. It has been predicted that CD4 would stabilize stimulatory (antigenic) but not homeostatic (self) TCR-pMHC interactions. It remains unclear how a molecule with extremely low 3D (K_d_ > 2.5 mM) and 2D (K_d_ ~ 4800 molecules/μm^2^) affinity for MHCII could achieve this, however (75). CD4 has a negligible effect on the TCR-pMHCII interaction (76, 77). On the other hand, CD4 forms a rather stable unit with Lck kinase (7), and the TCR/CD3 complex lacks enzymatic activity. Therefore, the signaling capacity and the ability to localize to MHCII-rich areas of the IS must determine CD4 function in T cells.

## CD4 in the Immunological Synapse

Varying levels of CD4 were reported to accumulate in the IS between T cells and the APCs that contain agonists (78–80). These discrepancies were probably caused by the employment of different experimental conditions and the sensitivity of applied imaging techniques. CD4 relocalization to IS matches that of TCRs (78) in terms of timing, but CD4 may have faster kinetics (79). Whereas, TCRs and signaling molecules accumulate in the center of the IS upon strong stimulation (Figure 2A), CD4 was found to distribute evenly throughout the IS or preferentially locate to the periphery of the IS [>3 min; (78)]. Importantly, CD4 relocalization to the contact site with MHCII-expressing cells is an antigen-independent process (79). Unlike with TCRs, the presence of antagonist does not prevent CD4 relocalization toward the APC. These results and new observations from Kuhns and colleagues (81) indicate that CD4 moves in T cell membranes independent of TCR/CD3 complex and does not pre-associate with TCR in unstimulated cells.

At the plasma membrane, CD4 is strongly associated with Lck (7, 82). Therefore, CD4 localization to the IS results in Lck accumulation therein (68, 83). Lck shows a delayed association with the IS in CD4-knockout T cells, which in turn delays phosphorylation of the Lck activation site (residue Tyr_394_ in mouse) and reduces CD4-knockout T cells responsiveness to antigens (83). On the other hand, phosphorylation of activatory tyrosine in Lck is not crucial to T-cell activation because anti-CD3ε antibodies do not induce such phosphorylation but can stimulate T cells (82, 84, 85). This can be explained by the modest increase in Lck activity (two- to three-fold) upon phosphorylation of the activating tyrosine. CD4 thus delivers the crucial kinase to the site of TCR triggering and enables its full activation to maximize the sensitivity of T cells toward rare and weak antigens [*Model 2* of the CD4 function in T cells - Figure 3; (62)]. Lck kinase activity does not affect CD4 accumulation in the IS because the Src-family kinase inhibitor (PP1) does not prevent localization of CD4 to the contact site with APCs (79).

## Antigen-independent Role of CD4 in T Cells

CD4 was originally described as an adhesion molecule that enhances the contact between T cells and APCs (86, 87). In their pillar work, Doyle and Strominger found a direct correlation between the extent of cell-to-cell adhesion and the level of MHCII and CD4 expression. Using a monolayer of CD4-expressing fibroblasts and Raji B cell line, which expresses high levels of MHCII, they eliminated the possibility that TCR or other T cell-specific molecules are involved in the interaction (86); observing no adhesion of the cells that did not express MHCII to CD4^+^ cells, thus confirming the specificity of that interaction. However, using surface plasmon resonance assays, other researchers have shown that CD4 binds the MHCII molecule with an extremely low 3D affinity [see above; (75, 88)]. This is further supported by the results of 2D binding studies on MHCII-expressing cells and lipid bilayer-anchored extracellular domains of CD4 (as well as of CD2 to allow cell adhesion). In agreement with the adhesion studies (86), this binding is specific because the MHCII non-expressing cells did not bind to CD4 on supported planar bilayers (75). CD4 very weakly bound to MHCII (approaching the detection limit of the method) according to an adhesion frequency assay with micropipette-attached interacting cells (77). Therefore, it is unclear how CD4 facilitates adhesion between coreceptor- and MHCII-expressing cells.

The way to explain the ability of CD4-MHCII interaction to facilitate both cell-to-cell adhesion and the antigen-independent accumulation of coreceptors at contact sites with MHCII-expressing cells can be the organization of these molecules in higher-order structures. Multimerization enhances the avidity of the TCR-MHC interaction (89–91). Similarly, the multivalent interaction of CD4 and its ligand can provide this interaction with the appropriate strength. In this direction, it was suggested that palmitoylation targets CD4 to membrane lipid domains, called lipid rafts (55). Because the support for the existence of these domains in living cells remains inconclusive (92, 93), future studies must determine whether CD4 is associated with such entities and, if so, with what kinetics. Alternatively, CD4 can form large oligomers in unstimulated T cells (94). These oligomers must be disassembled upon stimulation because it is unlikely that such large structures are associated with the TCR/CD3 complex or with TCR microclusters. CD4 associates closely (< 5 nm distance, as determined by Forster resonance energy transfer, FRET) with the TCR/pMHC complex upon stimulation (68, 71, 79). Whereas, biochemical and functional data indicate the existence of CD4 dimers (or higher order oligomers) in unstimulated T cells (94, 95), direct observations of fixed or living cells using FRET has provided conflicting data (57, 96, 97). In these studies, FRET values are very low compared to the dimeric controls, which indicates either that only a small fraction of CD4 is oligomeric or that these structures are highly unstable. Moreover, the FRET-based characterization of CD4 oligomers may suffer from the limitations of this method which cannot distinguish between clustered molecules and oligomers, except only when a protein is assembled in a stable structure and when the appropriate data analysis methods are used (98). Such conditions have not yet been applied in studies of CD4 oligomerization. Thus, direct proof of CD4 oligomerization in living cells is still missing.

Another possible way to increase availability of CD4 for multivalent interactions is the formation of molecular clusters with a high density of coreceptors. Such CD4 nanoclusters exist in both unstimulated and stimulated murine T-cell blasts (99) and unstimulated Jurkat T cells (100). We also previously found that clustering depends on the presence of intact CD4 extracellular domains and palmitoylation sites (100). Clustering in nanoscopic structures (average diameter of ~100 nm) allows for multivalent ligand binding and frequent rebinding (29), which can provide the CD4-MHC interaction with sufficient strength to stabilize the sites of the contact between CD4- and MHCII-expressing cells (86, 87). Shapes and molecular densities of CD4 clusters (99, 100) are similar to those of TCR and associated effector signaling molecules (25, 101). On the other hand, the 2D character of the applied analytical methods does not provide a full understanding of these structures.

**Figure 4:**
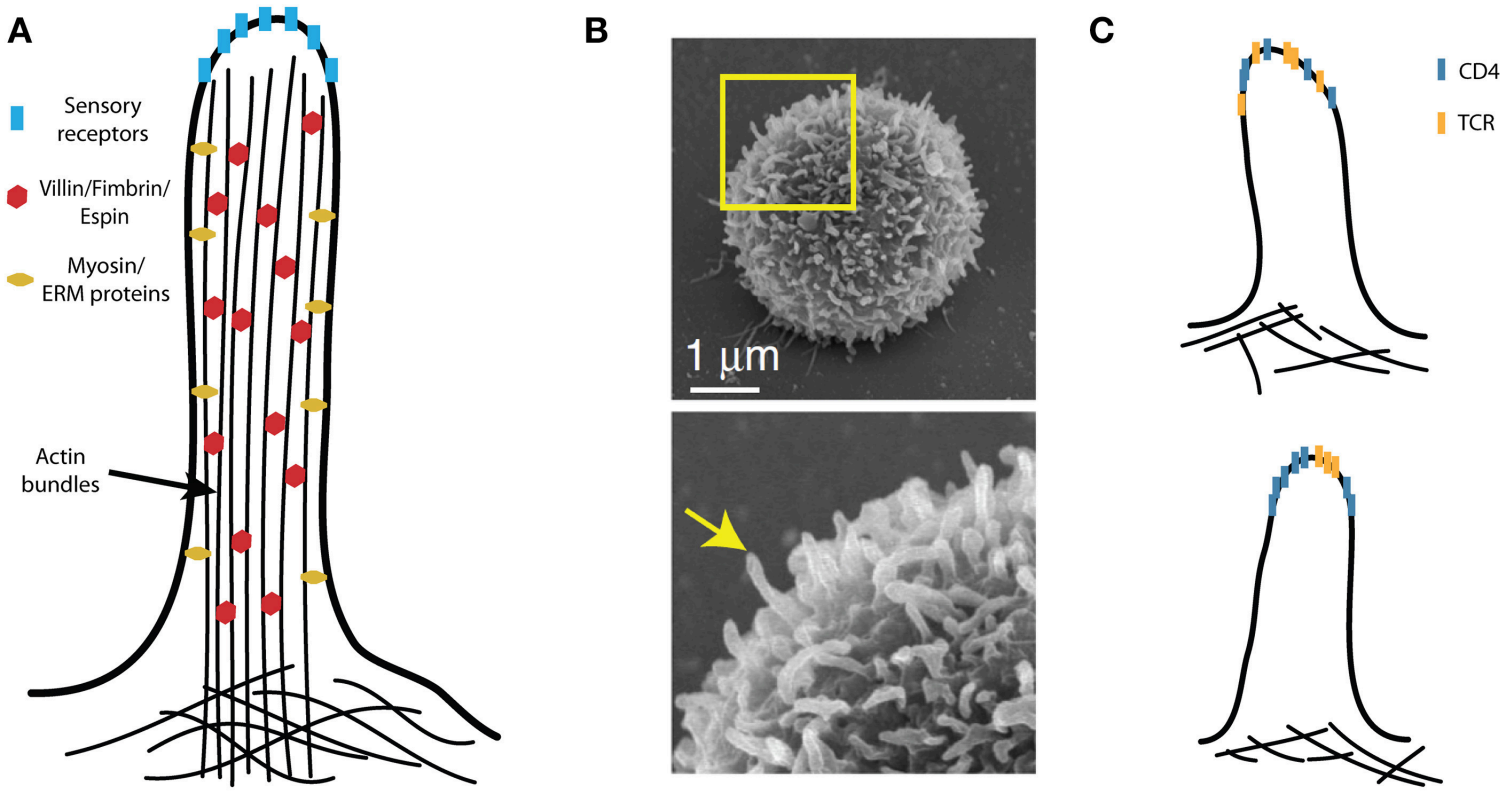
Microvilli in T-cell activation. **(A)** A schematic illustration of a microvillus with villin, fimbrin and espin internally cross-linking compact actin bundles, which tightly fill the microvilli. The plasma membrane is also closely associated with the actin bundles via the ezrin, redoxin, and moesin (ERM) proteins; the dynamics of microvilli involve specialized myosins. The tips of the sensory (i.e., hairy) cell microvilli accumulate critical receptors. **(B)** The peripheral T cells are covered by finger-like protrusions that are reminiscent of microvilli on sensory cells. **(C)** Molecular organization of CD4 with respect to other T-cell signaling molecules (e.g., TCR) on microvilli remains unknown. These molecules may be randomly distributed (upper panel) or assembled into specific domains (lower panel). **(B)** Is adapted from Kim et al. (104) licensed under Creative Commons **(CC BY 4.0)**.

More recently, TCRs and their effector molecules were found to accumulate in the tips of membrane protrusions that are reminiscent of *microvilli* (102, 103). Microvilli are finger-like plasma membrane protrusions with diameter ~100 nm; they are formed by cross-linked actin bundles that are tightly associated with membranes (Figure 4A). In sensory cells (e.g., hairy cells) or intestinal epithelial cells, these structures form extensive cell surfaces and accumulate selected receptors on their tips. The functions of microvilli in lymphocytes (Figure 4B) are less understood. Using scanning electron microscopy, microvilli were found to form primary contact sites with antigen-presenting dendritic cells (104, 105). The molecular details of this interaction have remained unknown until very recently (103). CD4 also accumulates in the microvilli of cultured T cells in a process that is regulated by the coreceptor association with Lck (106, 107). It is, therefore, possible that CD4 nanoclusters are indeed molecular assemblies of the coreceptor on the tips of the microvilli (Figure 4C). CD4 accumulation in the tips of the microvilli could explain its ability to facilitate adhesion between T cells and MHCII-positive cells.

Microvilli on dendritic cells interact with their counterparts on T cells during the antigen recognition (105). Biochemical and preliminary microscopy data indicate that MHCII accumulates in specialized membrane domains in a process that is regulated by members of the tetraspanins family, CD9 and CD63 (108–110). Tetraspanins CD9 and CD53 modulate the size and frequency of microvilli in leukocytes and epithelial cells (104, 111). However, it is unclear whether tetraspanins can enhance the sorting of MHCII to the tips of the microvilli; in addition, the organization of these structures on APCs must be characterized in the future.

## Conclusions and Future Perspectives

The results of 30 years of research indicate that CD4 has a dual function in peripheral T cells (and potentially in thymocytes). Firstly, it interacts with its ligand in an antigen-independent manner so as to induce contact between T cells and MHCII-expressing cells (***Model 3***; Figure 3). Second, CD4 interacts with pMHCII-TCR in an antigen-dependent manner so as to deliver Lck kinase to the complex and thus enhance T cell sensitivity (***Model2***). These two roles of CD4 do not have to be mutually exclusive. A direct role of CD4 in stabilizing the TCR-pMHCII interaction (***Model 1***) is not accepted any longer (62).

The antigen-independent function of CD4 is less understood. Its localization to microvilli (106) - as well as the evidence that the microvilli are the primary contacts between T cells and APCs (103–105) – indicates that CD4 can function as a scanning machinery, thus allowing T cells to select for cells that have MHCII on their surface. This may help to target TCRs toward the places with the highest MHC density and thus avoid interactions with cells that lack the ligand. The nanoscopic 3D organization of MHCII on APCs remains unknown. A full molecular anatomy of a synaptic vesicle (which was purified from neurons) indicates extreme protein density and reveals specific functional distribution of molecules in these structures (112). We believe that the creation of a similar model of microvillar tips on T cells and APCs will help to answer several intriguing questions regarding the initial phase of T-cell activation.

Importantly, the contact sites formed between T cells and APCs should also be explored using super-resolution techniques that have recently been adapted for living cells, including stimulated emission depletion (STED), super-resolution optical fluctuation imaging (SOFI) and lattice light sheet microscopy (103, 113, 114). Such studies are needed to confirm whether microvilli dominate the T cell-APC contact site and to determine the function of microvilli in T-cell activation. Other forms of membrane protrusions, such as filopodia and membrane ruffles, may also participate in this process. If CD4 scans the surface of the surrounding cells for MHCII-rich areas, it will be very important to determine whether such interactions stimulate changes in the T-cell membrane topology or molecular architecture of T cell microvilli, as has been observed in mechanosensory cells (115). Such changes may predetermine the local environment that TCRs require for rapid but highly selective antigen-induced signaling.

CD4 is one of the most studied molecules in the human body. This is mainly because it facilitates the infection of T cells with HIV-1. On the other hand, its function in various subsets of peripheral T cells remains poorly understood. New technologies that enable high-detail imaging of cellular structures provide previously unexplored ways to resolve such long-neglected topics. Using these techniques can lead to a better understanding of multifaceted role of CD4 in peripheral T cells and, potentially, in other CD4-expressing cells.

## Author Contributions

All authors listed have made a substantial, direct and intellectual contribution to the work, and approved it for publication.

### Conflict of Interest Statement

The authors declare that the research was conducted in the absence of any commercial or financial relationships that could be construed as a potential conflict of interest.
